# Porelis^TM^, a standardized purple tea extract, improves adipocyte functionality by regulating the leptin and adiponectin levels in high-fat diet-induced C57BL/6 mice

**DOI:** 10.22038/ijbms.2025.84192.18209

**Published:** 2025

**Authors:** Heggar Venkataramana Sudeep, Kuppam Sundeep, Karempudi Venkatakrishna, Amrutha Raj, Ranganath Bhumika, Thammatadhahalli Parameshwarappa Prasanna Kumar, Kodimule Shyamprasad

**Affiliations:** 1 R&D Center for Excellence, Vidya Herbs Pvt Ltd., #14A, Jigani I phase, Bangalore- 560 105, Karnataka, India

**Keywords:** Adipocyte differentiation, GHG, Leptin, Purple tea, Weight loss

## Abstract

**Objective(s):**

Purple tea variety of *Camelia sinensis *(L.) Kuntze (Theaceae), with its unique presence of anthocyanins and 1,2-di-*O*-galloyl-4,6-*O*-(S)-hexahydroxydiphenoyl-β-D-glucose (GHG), has enormous potential in the weight management. Here, the weight loss mechanism of a standardized purple tea hydroalcoholic extract (Porelis^TM^, 3-5% GHG) is demonstrated in high-fat-diet-induced mice.

**Materials and Methods:**

Male C57BL/6 mice (20±2 g) were divided into five groups (n=8). In a 12-week study, the control group was given a normal diet, and the other groups were given HFD (60 % kcal from fat) for 12 weeks. The treatment groups received Porelis at 25, 50, and 100 mg/kg body weight, PO.

**Results:**

Porelis treatment markedly reduced the body weight, fat pad weights, hepatic lipid accumulation, and oxidative stress in the HFD mice. The adipogenic markers, CCAAT/enhancer binding protein alpha (C/EBPα), Peroxisome proliferator-activated receptor gamma (PPAR-γ), and Sterol Regulatory Element Binding Protein 1 (SREBP1) were down-regulated in Porelis-treated mice compared to HFD model group (*P*<0.0001). Porelis treatment reduced the serum leptin level (*P*<0.0001) while increasing the adiponectin (*P*<0.01) in HFD mice. The circulating trimethylamine N-oxide (TMAO) (*P*<0.0001) and insulin (*P*<0.001) levels were significantly reduced in the Porelis groups compared to the HFD-alone group. In addition, Porelis markedly inhibited the *in vitro* pancreatic lipase activity (IC_50_ = 223.3 µg/ml).

**Conclusion:**

Porelis exerts antiobesity activity in mice by regulating adipogenesis and energy homeostasis. Our data encourages further validation of Porelis-mediated weight loss effects in human subjects.

## Introduction

Obesity, characterized by the excess fat accumulation in the body, is a global health concern affecting the world population. The causes of obesity include environmental and genetic factors and the imbalance in energy consumption and expenditure (1). Adipocyte functionality is compromised to a significant extent in obesity, leading to hyperplasia and hypertrophy. Further, adipocyte dysfunction can result in insulin resistance, inflammation, and other secondary health complications, including non-alcoholic fatty liver disease (NAFLD), cardiovascular diseases, and hypertension (2). 

Weight management strategies include lifestyle changes, dietary restrictions, and exercise (3). Available antiobesity drugs such as orlistat, sibutramine, and rimonabant have mild to severe side effects, such as insomnia, loss of appetite, high blood pressure, and heart attack (4). In this regard, plant-based functional supplements have recently gained popularity due to their limited or no side effects. 

Purple tea is a unique tea clone variety of *Camelia sinensis* (Fam. Theaceae) originally released by the Tea Research Foundation of Kenya (TFRK). This variety of *C. sinensis* is unique from the green and black tea preparations in having the presence of anthocyanins and 1,2-di-O-galloyl-4,6-O-(S)-hexahydroxydiphenoyl-β-D-glucose (GHG). Purple tea has comparatively less caffeine content than green and black tea. There are limited scientific studies on the pharmacological activities of purple tea preparations. Previously, it was demonstrated that purple tea anthocyanins could increase the anti-oxidant capacity in the mouse brain (5). Shimoda *et al*. studied the antiobesity effect of the purple tea extract in mice (6). Joshi *et al*. (7) reported the antioxidant and immunostimulatory activities of isolated anthocyanins from purple tea. More recently, da Silva *et al*. (8) studied the pancreatic lipase inhibition activity of the purple tea extract. There are no available studies on the comprehensive evaluation of the antiobesity effects of the purple tea extract. Based on the phytochemical content, it can be hypothesized that purple tea extract may have a multifaceted role as an antiobesity agent. In accordance with this hypothesis, we investigated the efficacy of a standardized purple tea extract in high-fat diet-induced obese C57BL/6 mice. 

## Materials and Methods

### Chemicals

Primary antibodies for CCAAT/enhancer binding protein alpha (C/EBPα, sc-365318), Peroxisome proliferator-activated receptor gamma (PPAR-γ, sc-7279), Sterol Regulatory Element Binding Protein 1 (SREBP1, sc-13551), and the specific secondary antibody were purchased from Santa Cruz Biotechnology, Inc. (Dallas, TX, USA). 2,2-diphenyl-1-picrylhydrazyl (DPPH) and sodium nitroprusside dihydrate were purchased from Himedia. 2,2-Azinobis(3-ethylbenzothiazoline-6-sulphonic acid (ABTS), 2,4,6-Tripyridyl-s-Triazine (TPTZ), N-(1-Naphthyl) ethylenediamine dihydrochloride, and orlistat were purchased from Sigma Aldrich Co. 

### Plant extract

The pulverized leaves (100 g) sourced from Mt. Kenya regions were extracted with 800 ml of 50% v/v ethyl alcohol in water containing 5 g of citric acid at 55–60 °C for three hours and cooled to room temperature. The extract was filtered by vacuum filtration. The extraction procedure was repeated thrice, using 600 ml of the above solvent solution and 1.5 g of citric acid each time. The collected filtrates were pooled and concentrated to dryness using a rotary flash evaporator under reduced pressure at 55–60 °C. The process was optimized for batch-wise consistency to get the final extract powder (Porelis^TM^) with a standardized content of GHG (3–5%). 

### Phytochemical characterization of Porelis

Porelis was quantitatively analyzed for catechins and GHG content using an HPLC-PDA detector. The separation was performed in a Shimpack scepter C18 column 120, 3 µm (4.6×150 mm) using gradient elution. The mobile phase comprised 0.05% trifluoroacetic acid in water (Solvent A) and acetonitrile (solvent B). The gradient elution consisted of 8–19.2% solvent B from 0.01 to 30 min, increasing solvent B to 50% from 30 to 33 min, and increasing again to 70% from 33 to 38 min. Then, solvent B was returned to the initial concentration within 40 min. The oven temperature was set at 30 °C with an injection volume of 10 µl. The detector wavelength was set to 230 and 270 nm for catechins and GHG, respectively. The limit of detection (LOD) and limit of quantification (LOQ) were 0.2 ppm and 0.8 ppm, respectively.

### Animal experiments

Five-week-old male C57BL/6 mice (20±2 g) were purchased from Biogen Laboratory Animal Facility, Bangalore, India. The animals were maintained in air-conditioned rooms with controlled temperature (22±3 °C) and relative humidity (30–70%) under a 12 hr light-dark cycle. Following 7-day acclimatization, the mice were randomized to 5 groups (n=8 per group): normal diet (ND), high-fat diet (HFD; Cat No. D12492, Research Diets, Inc., USA), HFD + Porelis (25 mg/kg/day), HFD + Porelis (50 mg/kg/day) and HFD + Porelis (100 mg/kg/day). Porelis was administered through oral gavage once daily at 25, 50, and 100 mg/kg for 12 weeks. The dose was chosen according to the previous study by Shimoda *et al*. (2015). The human equivalent dose based on the body surface area conversion factor ranges from 122–488 mg daily (9). The body weight, feed, and water consumption were recorded during the in-life phase. At the end of the experiment, the animals were fasted overnight and euthanized by overdose of isoflurane gaseous anesthesia. Blood samples were collected through a cardiac puncture, and serum was separated. The liver and fat pad (epididymal, perirenal, mesenteric, inguinal, brown adipose fat) were immediately removed, weighed, and photographed. The animal experimental protocol was approved by the Institutional Animal Ethics Committee (Approval No. VHPL/PCL/IAEC/08/2023) and followed the guidelines of the Committee for Control and Supervision of Experiments on Animals (CCSEA), India. During the experimentation, the animals were observed for any adverse events or clinical signs, and the humane endpoints were defined to reduce the pain/distress, if any. 

### Serum biochemical analysis

Serum total cholesterol (TC), triglycerides (TG), high-density lipoprotein (HDL) cholesterol, and low-density lipoprotein (LDL) cholesterol were determined by a Clinical chemistry analyzer (Unitron Biomedicals, Bengaluru, India). The serum non-specific enzymes aspartate aminotransferase (AST), alanine aminotransferase (ALT), and alkaline phosphatase (ALP) were analyzed using the assay kits from Elabscience Biotechnology Inc. USA. Serum leptin and adiponectin levels were analyzed by GENLISA^TM^ ELISA kits (Krishgen Biosystems, India) according to the manufacturer’s instructions. Serum insulin was assayed using an ELISA kit from Abbkine Scientific Co., Ltd., USA. 

### Trimethylamine N-oxide (TMAO) analysis in serum by LCMS method

Serum TMAO content was quantified using an Electrospray ionization source in positive mode by multiple reaction monitoring (MRM) method. The mobile phase consisted of 10 mM ammonium formate in 0.1% formic acid in water (solvent A) and acetonitrile (solvent B). The separation was performed using HI LIC column 250×4.6 mm with column oven temperature 40 °C maintained for isocratic elution (A:B;50:50) with flow rate of 0.8 ml/min and a run time of 10 min. The limit of detection (LOD) and limit of quantification (LOQ) were 0.5 ppb and 1.5 ppb, respectively. 

### Analysis of hepatic anti-oxidant status in HFD mice

The liver homogenates were assayed for superoxide dismutase (SOD), catalase (CAT), glutathione peroxidase (GPx) activities, reduced glutathione (GSH), and malondialdehyde (MDA) levels using ELISA kits (Elabscience Biotechnology Inc. USA), following the manufacturer’s instructions. 

### Histological examinations

The liver and epidydimal white adipose tissue (eWAT) samples were fixed in formalin solution and stained with Hematoxylin and eosin (H&E). The sections were observed under a microscope (Leica, Germany) equipped with a camera. The images were captured at × 200 magnification. 

### Quantitative Real-time PCR

Total RNA was extracted from the liver tissue (pooled samples from each group) using Trizol (Cat No. T9424, Sigma Aldrich Co., St. Louis, USA). cDNA was synthesized from 2 µg of RNA using a Hi-cDNA synthesis kit (Himedia Laboratories Pvt Ltd.). Real-time PCR was performed using SSO Advanced Universal SYBR® Green Supermix and CFX Opus 96 instrument (Bio-Rad). The PCR primer sequences used in this study are listed in Table 1. The real-time PCR conditions were as follows: 95 °C for 3 min followed by 40 cycles at 95 °C for 10 sec, 60 °C for 30 sec, and 72 °C for 30 sec. The relative gene expression was normalized to β-actin. 

### Western blot analysis

The epidydimal WAT was lysed in RIPA buffer and centrifuged at 10500 rpm, 4 °C for 20 min. The resultant tissue lysates were quantified for protein content using the Bradford protein assay kit (Bradford Laboratories Inc., Hercules, CA, USA). The equivalent concentration of proteins (50 µg) was separated using 12 % sodium dodecyl sulfate-polyacrylamide gel (SDS-PAGE) and transferred onto a nitrocellulose membrane. Tris-buffered saline containing 5% skimmed milk was used to block the non-specific binding. The membrane was then probed sequentially with primary and secondary antibodies, and the target proteins were detected using ECL substrate (Merk Millipore) and visualized on ImageQuant LAS 500 (GE Healthcare Life Sciences). 

### In vitro pancreatic lipase inhibition assay

Pancreatic lipase enzyme absorbs dietary free fatty acids in the intestinal lumen. The present study assessed Porelis for pancreatic lipase inhibition by the method described elsewhere (10). Briefly, an enzyme buffer was prepared by mixing 6 µl of porcine pancreatic lipase solution (pH 6.8) in MOPS (10 mM) and EDTA (1 mM) with 169 µl Tris buffer (100 mM Tris-HCl, 5 mM CaCl_2_, pH 7.0). The enzyme assay was performed by incubating 175 µl enzyme buffer and 5 µl of *p*-NPB solution (10 mM) in the presence or absence of different concentrations of Porelis (20-320 µg/ml) for 15 min at 37 °C. Orlistat (2.5-12.5 ng/ml) was used as a reference compound. The conversion of *p*-NPB to *p*-nitrophenol by lipase activity was quantified at 405 nm in a microplate reader. The % inhibition of lipase activity was calculated accordingly. 

The pancreatic lipase inhibition kinetics of Porelis (40, 80, and 160 µg/ml) were studied using 100, 200, and 400 µM concentrations of *p*-NPB. The maximum reaction velocity (V_max_) and Michaelis constant (K_m_) at different substrate concentrations were determined. The data were analyzed using a Lineweaver-Burk (L-B) double reciprocal plot. 

### In vitro anti-oxidant assays

As described elsewhere, the free radical scavenging potential of Porelis was determined using *in vitro* assays, such as DPPH radical scavenging (11), nitric oxide radical scavenging (12), ABTS radical scavenging (13), and hydroxyl radical scavenging (14) assays. 

### Statistical analysis

All the data were analyzed using GraphPad Prism 10.2.0 (GraphPad Software Inc.) and presented as mean ± standard deviation. The data were statistically analyzed using one-way ANOVA followed by Tukey’s test. *P*<0.05 were considered statistically significant. 

## Results

### HPLC analysis of Porelis

**Figure 1:**
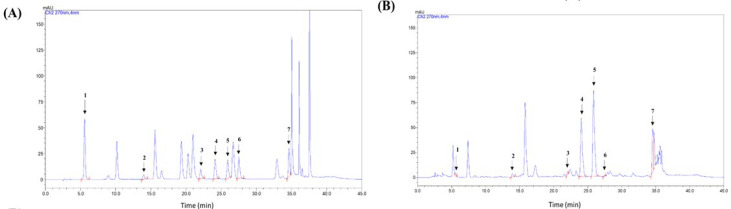
Representative chromatogram of phenolic acids detected in HPLC-PDA detector at 230 and 270 nm

Figure 1 shows the representative chromatogram of Porelis and the reference standard mix. The concentration of phenolic acids in Porelis is presented in Table 2. The extract contains GHG (35.53±2.00 mg/g), EGCG (61.27±4.067 mg/g), and epicatechin gallate (32.40±2.16 mg/g) as major phenolics. 

### Effect of Porelis administration on body weight and feed intake in HFD-induced mice

**Figure 2:**
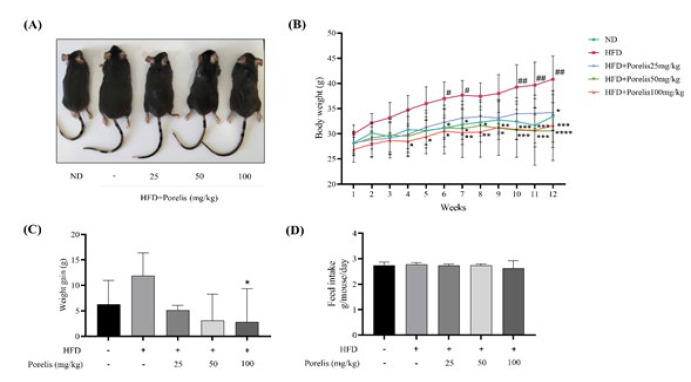
Effect of Porelis on body weight and feed consumption in HFD-induced C57Bl/6 mice

To examine the potential antiobesity effects of Porelis, mice were treated with 25, 50, and 100 mg/kg/day of Porelis simultaneously with HFD for 12 weeks. The body weight changes were recorded weekly during the study period (Figure 2). Figure 2A shows the representative photographs of the mice at the end of the study. As expected, the mice fed with HFD showed a significant increment in the body weight from week 6 until the end of the study (*P*<0.05) compared to the ND group (Figure 2B). However, the body weight of mice was markedly reduced in Porelis treated groups from the seventh week onwards, as compared to the HFD group (*P*<0.05). The HFD group exhibited a marked increase in body weight gain compared to the ND or Porelis-treated groups (Figure 2C). Interestingly, there was no significant change in daily feed consumption in the mice (Figure 2D).

### Effect of Porelis on fat pad weights, adipocyte size, and differentiation in HFD-induced obese mice

**Figure 3:**
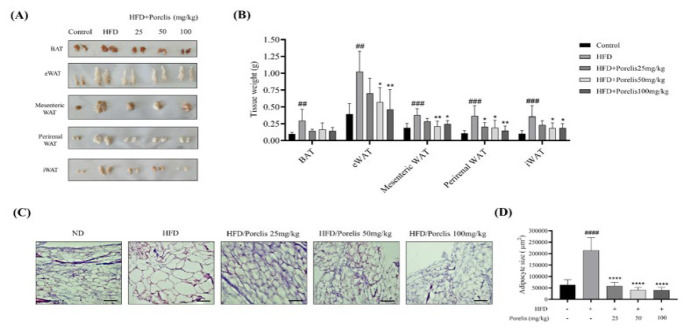
Effect of Porelis on fat accumulation and adipose tissue morphology in HFD-induced C57Bl/6 mice

To investigate the effect of Porelis on fat accumulation, we measured the different fat pad weights in mice (Figure 3A). It was observed that the brown adipose tissue (BAT) (*P*<0.01), epididymal (*P*<0.01), mesenteric (*P*<0.001), perirenal (*P*<0.001), and inguinal (*P*<0.001) white adipose tissues (WAT) were more significant in HFD group than those in the ND group (Figure 3B). Porelis administration significantly reduced the fat pad weights in HFD mice compared to the untreated HFD group (*P*<0.05). Histological examination of eWAT tissue samples revealed the characteristic changes in the HFD group relative to the ND group (Figure 3C). There was a clear increase in the adipocyte size of the HFD group compared to the ND group (*P*<0.0001). However, in the Porelis-treated mice, the adipocyte size and morphology were restored significantly as compared to the HFD group (*P*<0.0001) (Figure 3D). 

**Figure 4:**
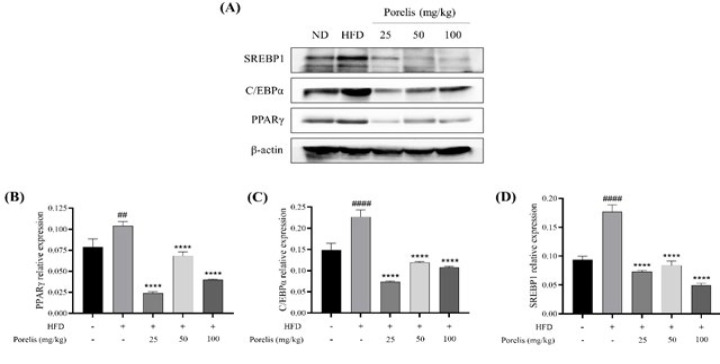
Effect of Porelis on adipogenic protein expression in eWAT of mice

Further, we examined the effect of Porelis administration on adipocyte markers in the eWAT tissue by western blot analysis (Figure 4A). The HFD mice showed a profound increase in the expression of adipocyte differentiation marker proteins viz., PPARγ (*P*<0.01), C/EBPα (*P*<0.0001), and SREBP1 (*P*<0.0001) compared to the ND group (Figure 4B-D). Porelis-treated groups considerably down-regulated the expression of these proteins as compared to the untreated HFD mice (*P*<0.0001). 

### Effect of Porelis on hepatic tissue architecture, lipid accumulation, and serum levels of liver function markers

**Figure 5:**
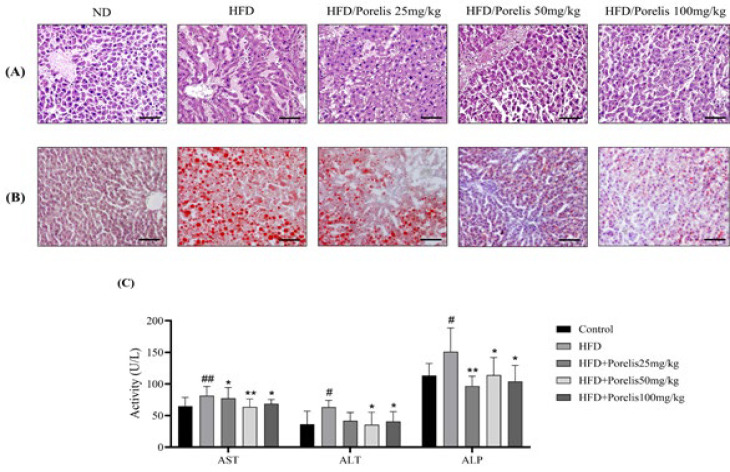
Effect of Porelis on the liver histomorphology and lipid accumulation in mice

The liver tissue samples were examined for histopathological changes using H&E staining (Figure 5A). The ND group exhibited normal cellular architecture. On the contrary, the HFD group showed obvious histopathological changes, such as hepatic steatosis, fatty vacuolations with necrosis, and inflammation. The Porelis treatment groups markedly restored the liver architecture and cell morphology compared to the untreated HFD group. 

Further, oil red O staining revealed that the HFD group showed a considerably higher lipid accumulation than the ND group. However, the Porelis treatment groups showed a reduced hepatic lipid accumulation compared to the HFD group (Figure 5B). 

Serum levels of AST, ALT, and ALP were increased significantly in the HFD group compared to the ND group (*P*<0.05) (Figure 5C). However, Porelis treatment significantly lowered the serum non-specific enzymes compared to the HFD group (*P*<0.05). These data suggest that Porelis exerts profound hepatoprotection in HFD mice. 

### Effect of Porelis on hepatic inflammation and anti-oxidant status in HFD-induced obese mice

**Figure 6:**
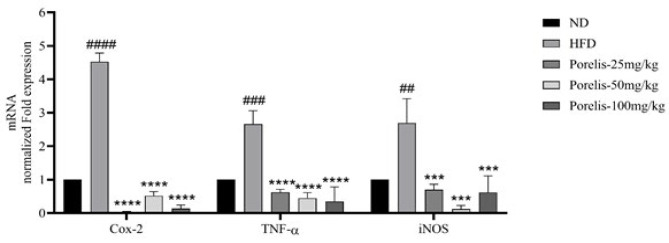
Effect of Porelis on hepatic gene expression of inflammatory markers

The hepatic gene expression of Cox-2 (*P*<0.0001), TNF-α (*P*<0.001), and iNOS (*P*<0.01) was increased by 4.53-fold, 2.67-fold and 2.7-fold, respectively, in the HFD group relative to the ND group (Figure 6). However, the Porelis treatment groups showed a considerably reduced expression of the inflammatory genes as compared to the HFD group (*P*<0.001). 

Further, we examined the hepatic anti-oxidant enzymes and oxidative stress markers (Table 3). As expected, the CAT and SOD levels were significantly reduced in the HFD group compared to the ND group (*P*<0.0001). Porelis administration dose-dependently restored the anti-oxidant enzyme levels compared to the HFD group (*P*<0.001). A similar trend was observed in the GPx analysis. However, the data were not significant. The hepatic oxidative stress in the HFD group was further evident from the elevated levels of MDA compared to the ND group (*P*<0.0001). The MDA level was significantly abated in the Porelis treatment groups compared to the HFD mice (*P*<0.0001). Also, Porelis at 100 mg/kg significantly increased the GSH level compared to the HFD group (*P*<0.05). 

### Effect of Porelis on serum lipid profile and TMAO levels in HFD-induced obese mice

**Figure 7:**
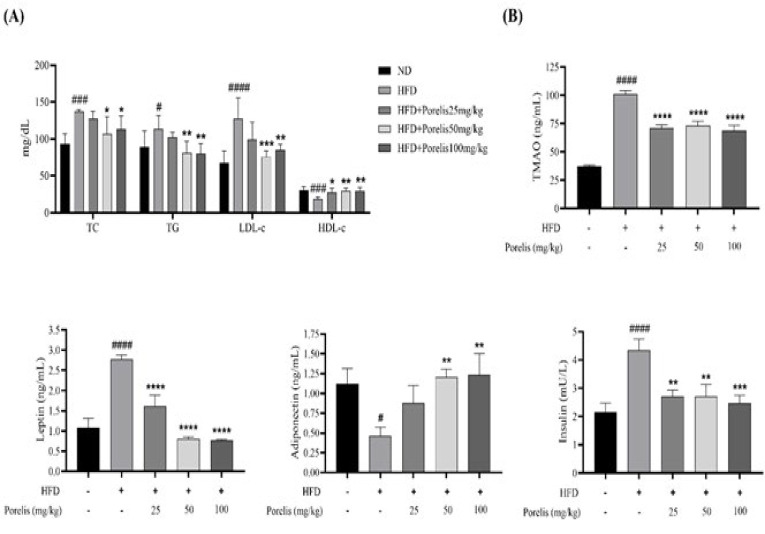
Effect of Porelis on serum biochemical parameters in HFD mice

To assess the effect of Porelis on the HFD-induced alterations in the lipid parameters, the serum samples were measured for TC, TG, HDL-c, and LDL-c (Figure 7A). As expected, the HFD group showed a significant increase in the serum levels of TC (*P*<0.001) and TG (*P*<0.05) compared to the ND group. Conversely, the Porelis treatment groups showed a considerable decrease in the TC (*P*<0.05) and TG levels (*P*<0.01) in a dose-dependent fashion compared to the HFD group. In addition, the HFD group mice showed significantly higher LDL-c and lower HDL-c than the ND group. Porelis treatment at 50 and 100 mg/kg significantly reduced the LDL-c levels in HFD mice compared to the untreated HFD mice. Also, the HDL-c levels were dose-dependently restored by Porelis administration at 25 (*P*<0.05), 50 (*P*<0.01), and 100 mg/kg (*P*<0.001) in HFD mice. 

The HFD group showed a significantly higher level of TMAO (2.72-fold, *P*<0.0001) compared to ND group mice (Figure 7B). However, Porelis treatment at 25, 50, and 100 mg/kg reduced the serum TMAO levels by 1.42-fold, 1.38-fold, and 1.48-fold, respectively relative to the HFD group (*P*<0.0001). 

### Effect of Porelis on leptin, adiponectin, and insulin

In the present study, we analyzed the serum markers of energy homeostasis (Figure 7C and Figure 7D). Compared to the ND group, HFD mice showed significantly higher serum leptin (*P*<0.0001) and lower adiponectin levels (*P*<0.05). Conversely, the Porelis treatment markedly reduced the serum leptin level at all the tested doses (*P*<0.0001) while increasing the adiponectin level significantly at 50 and 100 mg/kg doses, compared to the HFD group (*P*<0.01). We further assessed the serum insulin levels in mice. The HFD group exhibited a higher insulin level than the ND group (*P*<0.0001). Porelis treatment showed a comparatively decreased serum insulin level than the untreated HFD mice (*P*<0.01). 

### Effect of Porelis on pancreatic lipase activity

**Figure 8:**
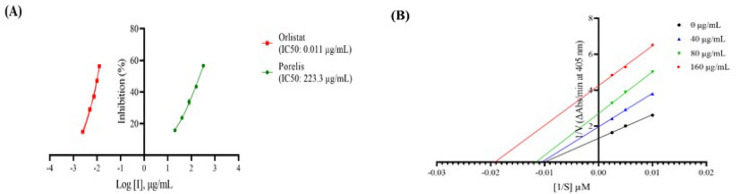
Effect of Porelis on pancreatic lipase activity

In this study, the *in vitro* pancreatic lipase activity was examined in the presence of different concentrations of Porelis or the reference compound orlistat. Porelis markedly inhibited the pancreatic lipase activity at the tested concentrations, with an IC_50_ of 223.3 µg/ml. As expected, the reference drug orlistat showed a strong inhibitory effect on enzyme activity (IC_50_: 0.01 µg/ml) (Figure 8A). 

The enzyme kinetics was studied at different concentrations of Porelis and the substrate. As shown in Figure 8B, the presence of Porelis at 40 (V_max_: 0.51, K_m_: 95.02), 80 (V_max_: 0.37, K_m_: 85.85) and 160 µg/ml (V_max_: 0.24, K_m_: 52.13) reduced both V_max_ and K_m_ values compared to the lipase activity in absence of extract (V_max_: 0.76, K_m_: 99.73). The kinetic data suggests that Porelis inhibits lipase activity in an uncompetitive manner.

### Free radical scavenging activity

**Figure 9:**
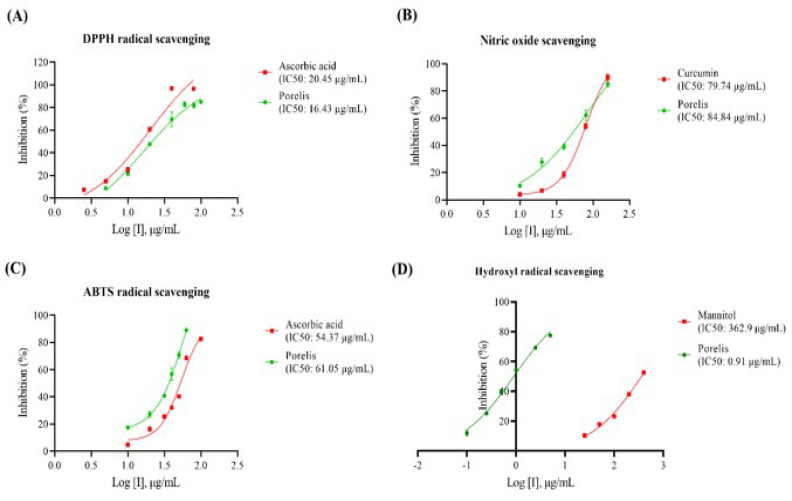
Effect of Porelis on* in vitro *free radical scavenging activities

The free radical scavenging ability of Porelis was studied using *in vitro* assays (Figure 9). In the DPPH scavenging assay, the IC_50_ value of Porelis was found to be 16.43 µg/ml, which was comparable to the reference compound ascorbic acid (IC_50_: 20.45 µg/ml). Porelis further exhibited profound anti-oxidant activity by nitric oxide and ABTS radical scavenging with respective IC_50_ values of 84.84 µg/ml and 61.05 µg/ml. The data were comparable to the reference compounds curcumin and ascorbic acid. Interestingly, Poralis showed significant hydroxyl radical scavenging activity with an IC_50_ value (0.91 µg/ml) considerably less than the reference compound mannitol (362.9 µg/ml). 

## Discussion

In the present study, we demonstrated the antiobesity effect of Porelis in HFD-induced C57BL/6 mice. It is well-reported that C57BL/6 mice are susceptible to diet-induced metabolic changes, including hyperlipidemia or dyslipidemia, thus resembling obesity or type 2 diabetes (15). A 12-week HFD consumption significantly increased the mice’s body weight compared to the ND group. However, simultaneous treatment of HFD mice with different doses (25, 50, and 100 mg/kg) significantly controlled the weight gain for up to 12 weeks. Interestingly, there was no significant change in the feed consumption between the Porelis-treated mice and the HFD group. These data suggest that the observed weight loss in Porelis-treated mice was not caused by appetite suppression. 

Obesity is characterized by excess fat accumulation in the fat depots and adipocyte dysfunction, resulting in adipocyte hyperplasia and hypertrophy (16). Regulation of preadipocyte differentiation is an effective strategy to treat obesity. In the present study, Porelis treatment considerably reduced the adipose tissue weights and adipocyte size in the mice, ameliorating the HFD-induced pathological manifestations in the adipose tissue. Further, we examined the effect of Porelis on adipocyte differentiation. PPARγ, C/EBPα, and SREBP1 are reported to be the key transcription factors that regulate adipocyte differentiation (17). Porelis administration significantly reduced the expression of these proteins in the eWAT tissue. The preclinical data align with our previous studies in 3T3L1 cells (18).

Obesity plays a crucial role in the development of simple steatosis and its progression into non-alcoholic steatohepatitis (NASH) (19). In the present study, the HFD mice showed significant lipid accumulation and pathological changes in the hepatocytes. However, the administration of Porelis restored the cellular architecture and considerably reduced the lipid accumulation in the liver. The hepatoprotective effect of Porelis was further evident from the reduced levels of serum non-specific enzymes, AST, ALT, and ALP, as compared to HFD mice. In addition, Porelis markedly reduced the hepatic inflammation and improved the anti-oxidant status. 

One of the major pathological manifestations of obesity is the occurrence of dyslipidemia (20). It has been demonstrated previously that long-term HFD consumption elevates the serum’s TC, TG, and LDL-c. Associated with the reduction in HDL-c, these alterations in the serum lipid profile would increase the risk factors for cardiovascular diseases (21). Porelis reduced TC, TG, and LDL-c while improving the HDL-c level in the serum. We further quantified the systemic levels of TMAO, a gut microbiome-dependent metabolite. The experimental and clinical evidence suggest that elevated levels of TMAO are associated with an increased risk of atherosclerosis and cardiovascular diseases (22, 23). In addition, there is a strong association between obesity and increased levels of TMAO (24). In line with these observations, the HFD mice showed a marked increase in the serum TMAO level compared to the ND group. Interestingly, Porelis at the tested doses significantly reduced the TMAO levels in mice. These results strongly suggest that ingestion of Porelis could mitigate the cardiovascular risk factors of obesity. 

Leptin and adiponectin, the adipokines produced in the adipose tissue, play a crucial role in energy homeostasis (25). The levels of these hormones are altered to a significant extent in obesity. Elevated leptin levels and reduced adiponectin levels result from adipose tissue dysfunction in obesity (26). In the present study, Porelis treatment significantly reduced the serum levels of leptin while improving the adiponectin level in HFD mice. Porelis-mediated reduction in the leptin level can be correlated with the decreased adipocyte size in the eWAT. Adipocyte dysfunction in obesity is associated with insulin resistance. In the present study, HFD mice showed higher serum insulin levels than the ND group. Porelis treatment considerably abated the serum insulin level in mice. Overall, these results suggest that Porelis could improve the adipocyte function via regulation of leptin and adiponectin and further enhance insulin sensitivity in HFD mice. 

Shimoda *et al*. (6) have demonstrated the antiobesity effects of the purple tea extract in diet-induced obese mice. However, during the study, the animals were given HFD for a shorter duration and administered with only a single dose of purple tea extract. In the present study, Porelis was orally administered at three doses (25, 50, and 100 mg/kg/day of Porelis equivalent to the human doses of 122–488 mg/day) with simultaneous feeding of HFD to the mice for 12 weeks. 

In the present study, Porelis, with its standardized contents of catechins and GHG, is demonstrated to possess a significant inhibitory effect against pancreatic lipase activity *in vitro*. Further, the kinetic data analysis showed that Porelis inhibited the respective enzyme activities through the uncompetitive mode of inhibition. 

The present study attempted to provide scientific evidence on the biomechanism of Porelis-mediated weight loss in mice. In this direction, we have gathered vital data on adipocyte differentiation and energy homeostasis, hepatic lipid accumulation, inflammation, serum lipid profile, and TMAO metabolism. However, the study has certain limitations. Firstly, the effect of Porelis on the body composition of HFD mice was not included in this study. Further, the study limits the quantification of TMAO, the gut microbial metabolite, and a marker of metabolic syndrome. It would be interesting to examine the remodeling of gut microbiota associated with the Porelis-mediated reduction of TMAO. The clinical relevance to the observed antiobesity effect of Porelis is further required to validate the experimental findings from this study. 

## Conclusion

The present study demonstrates that Porelis controls adipocyte differentiation via regulation of the transcriptional activation of PPARγ, C/EBPα, and SREBP1 in HFD mice. In addition, Porelis restores leptin, adiponectin, and insulin levels to improve energy homeostasis in HFD mice. The data from our study encourages Porelis to be a key ingredient in functional supplements for weight loss. However, human clinical trials are necessary to support the health claims and tolerability of Porelis. 

**Table 1:** List of primers used in high-fat diet-induced C57Bl/6 mice

Target gene	Forward primer (5'-3')	Reverse primer (5'-3')
Cox-2	TACTACACCAGGGCCCTTCC	TCAGGAAGCTCCTTATTTCCCTTC
TNF-α	ACAAGCCTGTAGCCCACG	TCCAAAGTAGACCTGCCC
iNOS	CTCTAGTGAAGCAAAGCCCAACA	CACATACTGTGGACGGGTCG
β-actin	CACTGTCGAGTCGCGTCCA	CATCCATGGCGAACTGGTGG

The values are presented as mean±SD of three independent experiments.

**Table 2:** Quantification of phenolic acids detected in Porelis by HPLC-PDA detector

Phenolic compound	Retention time (min)	mg/g extract at 230/270 nm
Gallic acid	5.585	0.5±0.006
Epigallocatechin	13.987	13.07±0.577
Epicatechin	22.106	3.83±0.252
Epigallocatechin gallate	24.165	61.27±4.067
GHG	26.723	35.53±2.000
Gallocatechin gallate	27.551	0.43±0.058
Epicatechin gallate	34.662	32.40±2.16

**Table 3:** Effect of Porelis administration on the hepatic anti-oxidant activity in HFD mice

Groups	Catalase U/mg protein	SOD U/mg protein	GPx U/mg protein	MDA µmol/g protein	GSH µmol/g protein
ND	160.3±5.15	228.4±15.37	19760±2242.00	1.16±0.05	3.11±0.12
HFD	121.5±3.89^####^	107.3±7.28^####^	17407±1278.00	3.18±0.24^####^	2.67±0.26
Porelis (25 mg/kg)	142.8±4.23^***^	148.0±2.10^***^	20615±717.20	1.79±0.16^****^	3.01±0.49
Porelis (50 mg/kg)	154.5±4.30^****^	151.3±2.38^***^	22599±3346.00	1.20±0.13^****^	3.40±0.14
Porelis (100 mg/kg)	155.4±2.60^****^	156.7±3.74^***^	21019±1151.00	0.93±0.06^****^	3.44±0.24^*^

The values are mean±SD (n=3 of pooled samples). The data were analyzed using one-way ANOVA followed by a Tukey test. ####*P*<0.0001 vs the ND group. *P<0.05, ***P<0.001, and ****P<0.0001 vs the HFD group.

ND: Normal diet; HFD: High fat diet; SOD: Superoxide dismutase; GPx: Glutathione peroxidase; MDA: Malondialdehyde; GSH: Glutathione

(A) standard mix of seven authentic phenolic acids, (B) Porelis. 1: Gallic acid, 2: Epigallocatechin, 3: Epicatechin, 4: Epigallocatechin gallate, 5: GHG, 6: Gallocatechin gallate, 7: Epicatechin gallate.

(A) Representative images of mice from different experimental groups, (B) Weekly body weight changes in mice, (C) Mean body weight gain after 12 weeks of treatment, (D) Daily feed consumption. The values are mean±SD (n=6). The data were analyzed using ANOVA followed by the Tukey test. #*P*<0.05 and ##*P*<0.01 vs the ND group. *P<0.05, **P<0.01 and ****P<0.0001 vs the HFD group. ND: Normal diet; HFD: High fat diet

(A) Representative photographs of fat pads, (B) Fat pad weights, (C) H&E microscopic images of epidydimal WAT, magnification ×100. Scale bar: 100 µm. (D) Adipocyte size of epidydimal WAT. The values are mean±SD (n=6). The data were analyzed using one-way ANOVA followed by a Tukey test. ##*P*<0.01, ###*P*<0.001, and ####*P*<0.0001 vs the ND group. *P<0.05, **P<0.01, and ****P<0.0001 vs the HFD group. HFD: High fat diet; ND: Normal diet; eWAT: Epidydimal white adipose tissue

(A) Representative immunoblots showing the protein expression. (B-D) Densitometry analysis of the adipogenic transcription factors. The values are mean±SD (n=3). The data were analyzed using one-way ANOVA followed by a Tukey test. ##*P*<0.01 and ####*P*<0.0001 vs the ND group. ****P<0.0001 vs the HFD group. eWAT: Epidydimal white adipose tissue; HFD: High fat diet; ND: Normal diet

The liver tissue samples were processed for (A) H&E and (B) oil red O staining. Magnification ×100. Scale bar: 100 µm. (C) Serum levels of liver function markers. The data were analyzed using one-way ANOVA followed by a Tukey test. #*P*<0.05 and ##*P*<0.01 vs the ND group. *P<0.05 and **P<0.01 vs the HFD group. HFD: High fat diet; ND: Normal diet; H&E: Hematoxylin and eosin

The values are mean±SD (n=3). The data were analyzed using one-way ANOVA followed by a Tukey test. ##*P*<0.01, ###*P*<0.001, and ####*P*<0.0001 vs the ND group. ***P<0.001 and ****P<0.0001 vs the HFD group. HFD: High fat diet; ND: Normal diet

(A) Serum lipid profile. The values are mean±SD (n=6). (B) TMAO, (C) leptin, (D) adiponectin, and (D) insulin levels in mice. The values are mean±SD (n=3). The data were analyzed using one-way ANOVA followed by a Tukey test. #*P*<0.05, ###*P*<0.001, and ####*P*<0.0001 vs the ND group. *P<0.05, **P<0.01, ***P<0.001, and ****P<0.0001 vs the HFD group. HFD: High fat diet; ND: Normal diet

(A) Dose-response curves of percent inhibition versus log concentration. (B) Lineweaver-Burk plot analysis of the enzyme kinetics in the presence and absence of Porelis.

(A) DPPH radical scavenging, (B) nitric oxide scavenging, (C) ABTS radical scavenging, and (D) hydroxyl radical scavenging activities of Porelis were compared with respective standards. DPPH: 2,2-diphenyl-1-picrylhydrazyl; ABTS: Himedia. 2,2-Azinobis(3-ethylbenzothiazoline-6-sulphonic acid

## Data Availability

The data sets used and/or analyzed during the current study are available from the corresponding author upon reasonable request.

## Declaration

We have not used any AI tools or tecchnologies to prepare this manuscript.
